# Can an Extra-Uterine Pregnancy Terminate in Natural Delivery?

**Published:** 1889-12

**Authors:** 


F. E. DANIEL, M. D., EDITOR AND PUBLISHER.

THIS JOURNAL, by unanimous vote of the members, was made the Official Organ of the Austin District Medical Society.

COLLABORATORS:

*Dr. R. M. Swearingen, Austin.*

*Prof. B. E. Hadra, M. D., Galveston.*

*Prof. Geo. Cupples, M. D., San Antonio.*

*Dr. T. C. Osborn, Cleburne, Texas.*

*Dr E. J. Doering, Chicago.*

*Dr. E. J. Beall, Fort Worth.*

*Dr Odo Betz, Germany.*

*Dr. J. W. McLaughlin, Austin.*

*Dr. T. J. Tyner, Austin.*

*Prof. J. F. Y. Paine, M. D., Galveston.*

*Dr. R. H. L. Bibb, Mexico.*

*Dr. T. J. Bennett. Austin.*

*Dr. Bat Smith, Wharton.*

*Dr. E. Meierhof, New York.*

*Prof. W. B. Rogers, M. D., Memphis, Tenn.*

### CAN AN EXTRA-UTERINE PREGNANCY TERMINATE IN NATURAL DELIVERY?

At first blush the above question would strike most readers as absurd; in the light of the fact that even tubal pregnancies never go to term but result in rupture generally, not later than the third month,—collapse, and,—unless timely surgical aid is rendered, death. But Dr. S. T. Lowry, of San Antonio, a gentleman well and favorably known, a member of the Texas State Medical Association, a physician of repute, has recently published the report of a case (Courier Record of Medicine, Sept. 1889,) in which he declares that the fœtus was outside of the uterus, or what is the same thing, that the uterus and fallopian tube were empty, on examination while the woman was in labor; and that at a later examination, the next day, perhaps, or the next, his finger came in contact with the caput succedaneum, in the womb, and that the woman gave birth to a small living child; the placenta not coming away, he introduced his hand and removed it. Without saying so, for the Doctor does not say what kind of an extra uterine pregnancy it was, he assumes that the fœtus, with all its membranes was contained in a sac of some kind, outside of the womb, and that it entered the womb through the right fallopian tube; at least, that seems the rational inference from reading his paper. And Doctor Lowry's statements are entitled to respectful consideration.

In this issue we present a letter from Dr. Cross, a colleague of Dr. Lowry, in which he calls into question the correctness of Dr. Lowry's diagnosis, and asks some questions which, it seems to us, are pertinent. Unless Dr. Lowry is greatly mistaken in his judgment and diagnosis, his case is certainly unique, and his observation a remarkable one; and his courage, to call it by that name, in passing his hand through the uterine cavity, which he says was about large enough to contain the fist, into and through the fallopian tube (?) up, and into what must have been the free peritoneal cavity;—was certainly of a rare kind. But, as Dr. Cross asks, to what was the placenta attached? What contained the amniotic fluid? was there a cyst? what "cavity" was it into which he passed his hand, if not the free abdominal peritoneal cavity; and, as there must have been some hemorrhage on separating the placenta, what became of it? As said in the beginning, the question forming the caption of this article would appear absurd, natural delivery of an extra uterine fœtus, i. e., an abdominal pregnancy,—for it seems that "tubal" and "ovarian" are excluded,—even should gestation by any chance, go on to term, would appear a physical impossibility; but here is a gentleman of unquestioned veracity, a physician of perhaps twenty years practice, a man of good judgment and whose diagnostic powers we have never before heard called into question, who seriously asserts that an extra uterine pregnancy of some sort, not tubal or interstitial, actually went on to full term, and there was delivered, presumably through the fallopian tube, a living child! Our reading on this subject has not been very extensive, and our observation less, but so far as we know, Dr. Lowry's case is without a parallel in medical literature, ancient or modern; a wonderful case.

It seems there was no symptom indicating rupture of the tube, at any time, and there must have been rupture before it was possible for the product of conception to drop into the peritoneal cavity.

In rare cases this has occurred, a tube burst, the fœtus falling into the abdomen has escaped destruction and gone on to term, the placenta attached to some of the abdominal viscera; in such cases labor occurs, but nothing was ever "born"—the product being removed then or later, by section. But the removal of the placenta in such cases is attended with the utmost danger, and the boldest dare not do it, even with the aid of clamps or ligatures. What, then, would be the result of introducing the hand, even were it possible to pass an arm through the fallopian tube, and tearing away a placenta attached, perhaps, to omentum, or bladder, or intestine? Sure and speedy death. We cannot help thinking Dr. Lowry was mistaken.

